# Basic mathematical errors may make ecological assessments unreliable

**DOI:** 10.1007/s10531-017-1418-5

**Published:** 2017-08-24

**Authors:** P. R. Lintott, F. Mathews

**Affiliations:** Hatherly Laboratories, Biosciences, College of Life and Environmental Sciences, University of Exeter, Prince of Wales Road, Exeter, EX4 4PS UK

**Keywords:** Chiroptera, Conservation management, Environmental impact assessment, Mitigation, Statistics

## Abstract

Environmental impact assessments (EIAs) are used globally as the evidence-base for planning decisions, yet their efficacy is uncertain. Given that EIAs are extremely expensive and are enshrined in legislation, their place in evidence-based decision making deserves evaluation. The mean is the most commonly used summary statistic in ecological assessments, yet it is unlikely to be a good summary where the distribution of data is skewed; and its use without any indication of variability can be highly misleading. Here, using bats as an example, we show that EIAs frequently summarise these data using the mean or fail to define the term ‘average’. This can lead to the systematic misinterpretation of evidence which has serious implications for assessing risk. There is therefore a pressing need for guidance to specify data processing techniques so that planning decisions are made on a firm evidence-base. By ensuring that data processing is systematic and transparent it will result in mitigation decisions and conservation strategies that are cost-effective and proportionate to the predicted degree of risk.

## Commentary

Environmental Impact Assessments (EIAs) are relied on as the evidence-base for planning decisions, yet their efficacy is uncertain (Sutherland et al. 2006). Technological advances mean that the quantity of ecological data that can be gathered for appraisal is increasing rapidly. Despite the advantages of gathering more evidence, this information must nevertheless be summarised to permit easy interpretation by decision makers and other stakeholders. Errors at this stage can therefore lead to the systematic misinterpretation of evidence. Here, using bats as an example, we demonstrate that the misapplication of basic statistics can have important implications for assessing risk.

**Fig. 1:**
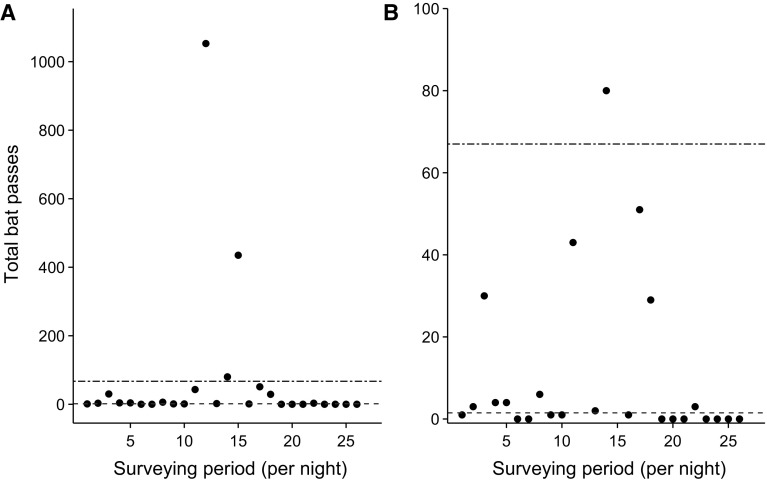
Bat activity recorded by a static bat detector at a moorland wind farm site during the National Bats and Wind Turbines project (Mathews et al. 2016). **b** is the same dataset as **a** however does not show the two nights of high bat activity so the difference between the mean (*blue line*) and median (*red line*) is clearly seen. (Color figure online)

Given the high legal protection afforded to bats (e.g. Europe, Eurobats 1991; North America, Endangered Species Act 1973), detailed pre-construction risk assessments are frequently undertaken. The rapid technological development in acoustic recorders has made the use of static surveys, where automated bat detectors are left out for multiple nights, commonplace during these assessments in an attempt to capture ‘average’ or ‘peak’ bat activity, and so permit an inference of risk. The summary statistic most commonly used to report bat activity in professional ecological assessments is the mean. For example, in a sample of 30 randomly selected UK ecological assessments (EcIAs) considering bats, 17 (57%) cited the mean, whilst the remainder (43%) failed to define the term ‘average’. In European guidelines for the surveillance and monitoring of bats (EUROBATS; Battersby 2010 **)** there is guidance for calculating ‘annual means’ of bat populations, whilst in South Africa the estimated collision risk to bats at wind farms is based on the mean number of bat passes per hour (Sowler et al. 2016). Yet the mean is unlikely to be a good summary of data that are skewed or highly clustered. For example bat activity is known to vary depending on factors such as temperature, seasonality, wind speed, and insect availability (Fischer et al. 2009); and for rare species where few data can be collected, the estimated mean will depend heavily on a small number of data points. For example, independent assessments showed that nightly bat activity did not conform to statistical normality at any of the 46 wind farm sites surveyed as part of the UK National Bats and Wind Turbines project (Mathews et al. 2016). Upland moorland is a relatively poor habitat for bats but may see occasional peaks in bat activity during periods of low wind speed and high temperatures when foraging opportunities become profitable. Taking the mean across multiple nights of bat activity may therefore result in an overinflated estimate of bat activity (Fig. 1) potentially leading to the imposition of costly and unnecessary mitigation measures. Conversely, heterogeneous lowland habitats are frequently used by bats but the high temporal and spatial variability in bat activity (Skalak et al. 2012) means that nights of zero activity are still likely to occur. Presenting the mean bat activity on these occasions would underestimate habitat usage leading to insufficient mitigation actions occurring.

The presentation of inappropriate summary statistics also hinders the ability of research scientists to use the data on which the EcIA is based (e.g. to assess the effectiveness of mitigation for major road developments). A good environmental assessment should disclose all relevant information to allow the significance of the environmental effects of the proposed development to be determined (Elkin and Smith 1988). It is evident that presenting just the mean number of bat passes fails to meet this standard. We therefore suggest that in many circumstances presenting the median and inter-quartile range would give a better understanding of usual activity/abundance. Similarly, presenting information on the relative frequency of occurrences of ecological importance (e.g. the number of nights a rare bat is encountered at a proposed development site) would give a better understanding of the likely consequences of the development than just presenting the mean. Therefore, there is a pressing need for future guidance to specify data processing techniques, so that both policymakers and practitioners are fully aware of how summarised data is analysed and presented, and to ensure that raw data are archived for future scrutiny. By ensuring that data processing is systematic and transparent it will also increase the reliability of comparisons of bat activity between sites which will ensure that planning decisions, mitigation decisions, and conservation strategies are cost-effective and proportionate to the predicted degree of risk to bats.
